# Concrete Protective Layer Cracking Caused by Non-Uniform Corrosion of Reinforcements

**DOI:** 10.3390/ma12244245

**Published:** 2019-12-17

**Authors:** Lu Zhang, Ditao Niu, Bo Wen, Daming Luo

**Affiliations:** 1 Department of Civil Engineering, Xi’an University of Architecture & Technology, Xi’an 710055, China; zhanglu@live.xauat.edu.cn; 2 State Key Laboratory of Green Building in Western China, Department of Civil Engineering, Xi’an University of Architecture & Technology, Xi’an 710055, China

**Keywords:** non-uniform corrosion, radial displacement, corrosion ratio, FEM, stress distribution

## Abstract

The volume expansion of reinforcement corrosion products resulting from the corrosion of steel reinforcement embedded into concrete causes the concrete’s protective layer to crack or spall, reducing the durability of the concrete structure. Thus, it is necessary to analyze concrete cracking caused by reinforcement corrosion. This study focused on the occurrence of non-uniform reinforcement corrosion in a natural environment. The characteristics of the rust layer were used to deduce the unequal radial displacement distribution function of concrete around both angular and non-angular bars. Additionally, the relationship between the corrosion ratio and the radial displacement of the concrete around the bar was established quantitatively. Concrete cracking due to the non-uniform corrosion of reinforcements was simulated using steel bars embedded in concrete that were of uneven displacement because of rust expansion. The distribution of the principal tensile stress around the bar was examined. A formula for calculating the critical radial displacement at the point when cracking began was obtained and used to predict the corrosion ratio of the concrete cover. The determined analytical corrosion ratio agreed well with the test result. The effect factor analysis based on the finite element method indicated that increasing the concrete strength and concrete cover thickness delays concrete cracking and that the adjacent rebar causes the stress superposition phenomenon.

## 1. Introduction

Many factors can reduce the durability of a reinforced concrete (RC) structure. Among them, the corrosion of internal reinforcement materials, which is known as reinforcement corrosion, is the primary cause. The volume expansion caused by the corrosion of reinforcing bars in concrete leads to cracking or spalling in the concrete protective layer as corrosion develops, followed by durability failure of the concrete structure [1,2]. Corrosion of steel bars leads to a decrease in the bonding force with concrete [3,4,5,6]. Steel corrosion has caused incalculable damage to several RC structures, necessitating their repair or removal. Thus, the structural damage caused by steel corrosion has become a global disaster risk concern. Failure due to steel corrosion is a complex process, and several problems remain to be solved.

After the reinforcement has corroded, an extrusion force on the concrete around the reinforcement is induced by the corrosion products. Under this reinforcement rust expansion force, the concrete cracks further. As the degree of rust corrosion increases, the cracks gradually expand toward the surface of the protective concrete layer. Several theoretical studies have been conducted on the reinforcement rust expansion force. According to an elastic analysis in the cracking process of corroded products, Bazant et al. [7] proposed three types of concrete failure. Weyers [8,9] performed a mechanical analysis of the cracking process by considering the diffusion of rust products into the concrete pores around the steel bars. Additionally, a circumferential stress formula based on elastic mechanics for the uncracked part of a cylinder with a unit length radius was proposed. Zhao et al. [10,11] analyzed the mechanical behavior of both the concrete protective layer and the rust layer, using elastic theory to obtain the deformation coordination relationship between the solidified soil and the rust layer. According to the theory of damage mechanics and mechanics of elastic solids, they introduced a damage variable to establish a model for the rust swell cracking of a partially cracked protective concrete layer. Li et al. [12] employed fracture mechanics to analyze the stress and strain in concrete. Their model determined the relationship between the width of the cracks in the protective layer and the corrosion depth of the reinforcement. The model of the main theoretical basis is the mechanics of elastic solids and fracture mechanics. The calculation process was complicated and the field measured data were needed, so the engineering application is relatively difficult.

Experimental studies on the rust-driven cracking of a concrete protective layer focus mainly on two topics: (1) the time from the rusting of the steel bar to the point when cracks appear on the concrete surface or the corrosion loss at the time of cracking on the concrete surface, and (2) the development of a law to predict the width of cracks on the concrete surface resulting from reinforcement corrosion. Numerous scholars have performed experiments to establish empirical models for the concrete cracking time and crack width. Andrade et al. [13] performed accelerated corrosion tests on four test blocks with different rebar positions, rebar diameters, and protective-layer thicknesses under the influence of different electric fields. Assuming the uniform corrosion of the steel bars, the corrosion depth of steel at a given time was calculated according to Faraday’s law, using the energizing current and the energization time. Alonso et al. [14] adopted similar test equipment at different current intensities to establish the relationship between the corrosion depth and the current intensity. Oh et al. [15] used the strain value to judge concrete cracking, determining that the initial crack appears on the concrete surface of the protective layer when the monitored strain reaches the value of the concrete’s cracking strain. Thus, the relationship between the corrosion rate of the steel reinforcement and the cover thickness was established by determining the point at which the concrete surface of the protective layer cracks. Song et al. [16] investigated the corrosion rate of rust expansion cracking on admixture concrete using accelerated corrosion tests under an electric field. They established a mathematical model to describe the steel corrosion rate in the case of a concrete protective layer under different environmental conditions, with consideration of the similarity between rapid electrochemical corrosion and the natural corrosion process.

Various factors contribute to the cracking of concrete structures due to corrosion expansion, but these factors cannot be comprehensively analyzed using empirical formulas. Previous research findings cannot be applied to other rust crack tests based on a small subset of factors. Therefore, in this study, a steel corrosion rate model was established for the situation in which a protective concrete layer is cracked, with consideration of various factors, such as the concrete cover thickness, water–binder ratio, and diameter of the steel bar.

Failure of concrete under the action of the steel rust expansion force is simulated using finite element software or a custom computer program developed for this purpose. Val et al. [17] studied the rust filling process in the free expansion stage of corrosion using a finite element model of a two-dimensional (2D) plane with holes. The difference between the experimental corrosion value at the time of cracking and the corresponding numerical simulation results reflects the quantity of rust filling. Jang et al. [18] performed a numerical simulation to examine the concrete cracking caused by non-uniform corrosion. The diameter of the steel bar, the cover thickness, and the rebar diameter at the time of concrete cracking were determined by adjusting the loads, material parameters, and geometrical dimensions. Zhao et al. [19] analyzed the distribution model of a rust layer of varying thickness around a steel bar. As the basis of the displacement load in the finite element model, corroded RC specimens were prepared to facilitate the study of the non-uniform rust-driven cracking process. The stress field distribution of the concrete caused by the non-uniform corrosion of the internal steel bar, the development and distribution of the rust expansion crack, and the corrosion expansive force were obtained by a finite element model. Ožbolt et al. [20] used a three-dimensional (3D) numerical model for transient analysis of the processes after depassivation of reinforcement in concrete, which is relevant to the calculation of the corrosion rate. The model predicted that cracks do not influence the corrosion rate for the case where the only influence of the crack is on the rate at which oxygen can reach the steel. Du et al. [21] examined the cracking of the concrete protective layer caused by non-uniform reinforcement corrosion using a microscale numerical simulation. The effects of the reinforcement diameter, cover thickness, and reinforcement-position on the cracking of the concrete protective layer under non-uniform corrosion conditions were investigated. Zhang et al. [22] numerically simulated a non-uniform corrosion of reinforcement that causes concrete cracking in chloride-contaminated RC structures. The effects of the concrete cover thickness, rebar diameter, and rebar spacing on the failure patterns of the concrete cover and the crack propagation were examined, and limiting criteria for the cracking modes were established. Fahy et al. [23] considered the transport of corrosion products into pores and cracks in concrete when predicting corrosion-induced cracking in RC structures. The pressure-driven transport was studied using an axisymmetric thick-walled cylinder model and a network method. Zhen et al. [24] studied the effects of different factors on the crack initiation time and crack propagation by using the thermal simulation method with a 3D nonlinear finite element model. The results indicated that the types of corrosion products, thickness of the interfacial transition zone, and corrosion rate are the most significant parameters that affect the crack initiation time. Xi et al. [25,26] established a mesoscale mixed-mode fracture model for concrete structure cracking. The effect of aggregate randomness on the crack width development of the difference between the uniformity and non-uniformity of concrete structures is that comprehensive parameters of corrosion have been investigated and proposed. Yang et al. [27] performed a numerical prediction of the concrete crack width and developed a numerical method for predicting the concrete crack width for corrosion-affected concrete structures. Accurate prediction of the crack width and timely maintenance are important for the service life of RC structures.

At present, the standard approach for the simulation of the process of concrete cracking caused by corrosion expansion is the use of a 2D model with a circular hole inside the concrete. However, such a 2D model cannot be used to analyze the interaction between sections, fracture development, or concrete spalling after rust-driven cracking. Therefore, in this study, focusing on the non-uniform corrosion of reinforcement in natural environments, an unequal radial displacement distribution function for concrete around angular and non-angular steel bars was deduced using the characteristics of the rust layer. A mathematical model for the critical radial displacement at the point when the concrete protective layer cracks was obtained in accordance with the results of a numerical regression analysis. The relationship between the radial displacement and the steel corrosion rate was used to obtain a calculation model for the corrosion rate of steel at the time of cracking. The prediction model was used to estimate the cracking time of the protective layer. Finally, the non-uniform rust calculation model was validated by comparing the results of the numerical analysis with experimental results.

## 2. Radial Displacement Field of Concrete with Non-Uniform Corroded Steel

After the reinforcement materials begin to corrode, corrosion products exert an extrusion force on the concrete around the reinforcement. Under this reinforcement rust expansion force, a radial displacement field is generated in the concrete surrounding the reinforcement. If this radial displacement field can be determined, the volume expansion of the rust product can be simulated via the displacement method. In a real-world application environment, the corrosion of steel in RC members is non-uniform. The concrete side near the protective layer tends to be badly corroded, while the other side exhibits the opposite pattern. The radial displacement field of the reinforcement in angular and non-angular regions can be determined using findings from previous research.

### 2.1. Non-Angular Area Reinforcement

**Figure 1:**
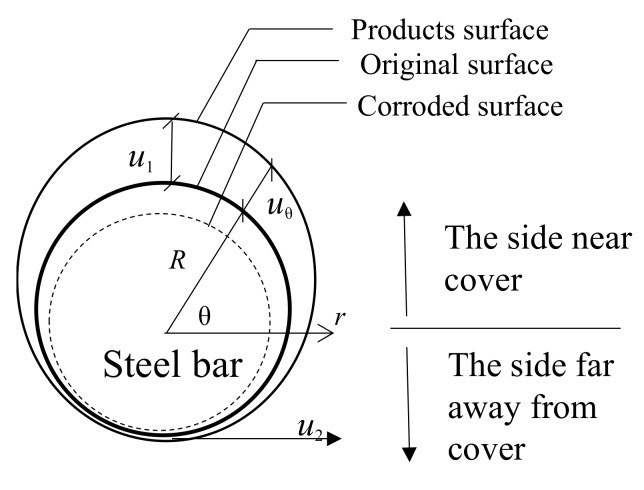
Radial displacement distribution model for non-angular area reinforcement.

Reinforcement corrosion is distributed such that the thickness of the rust layer is inversely proportional to the shortest distance to the concrete protective layer. A point closer to the concrete surface corresponds to a thicker rust layer on the rebar. An elliptical model of the rust layer’s outer contour line in a non-angular reinforcement area is proposed. Before the expansion, due to the corrosion of the reinforcing causes the concrete to crack, the reinforcement corrosion is distributed in a semicircle on one side of the concrete protective layer, and the rust layer on the other side of the semicircle is so comparatively small that it is ignored in the calculations [28]. However, after the cracks begin to appear in the concrete, the distribution model of the steel corrosion layer can be divided into two parts: an elliptical model situated near corresponding to one side of the concrete protective layer and a uniform corrosion model corresponding to the other side. The radial displacement distribution model for a non-angular reinforcement area is shown in Figure 1 and given by Equation (1).

(1) $$u(\theta )=\left\{\begin{array}{l} 0\leq \theta \leq \pi \;\frac{\left(r+u_{1})\cdot (r+u_{2}\right)}{\sqrt{{\left(r+u_{1}\right)}^{2}\cos^{2}\theta +{\left(r+u_{2}\right)}^{2}\sin^{2}\theta }}-r\\ \pi \leq \theta \leq 2\pi \;u_{2} \end{array}\right.$$

in which *r* is the original radius of the steel bar, *u*_θ_ is the corroded thickness at *θ* under polar coordinate, *u*_1_ is the maximum corroded thickness nearest the concrete cover (for the side located bar) or the maximum corroded thickness at the corner of the concrete specimen (for the corner located bar), and *u*_2_ is the corroded thickness at the side far away from concrete cover.

### 2.2. Angular Area Reinforcement

**Figure 2:**
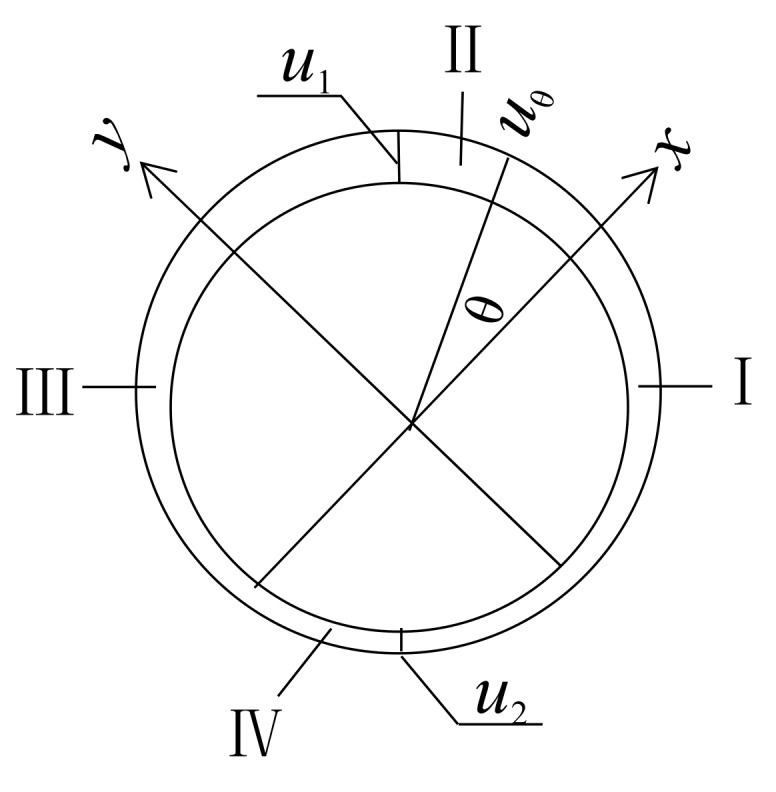
Radial displacement distribution model for angular area reinforcement.

Based on the results of a previous study [28], a double ellipse model for the reinforcement corrosion layer in an angular area is proposed. It is assumed that the shape of the rebar residual section is approximately elliptic. The radial displacement distribution model for angular area reinforcement is shown in Figure 2 and given by Equation (2).

(2) $$u(\theta )=\left\{\begin{array}{cc} -\frac{\pi }{2}\leq \theta \leq 0 & \frac{\left(r+u_{1})\cdot (r+u_{2}\right)}{\sqrt{{\left(r+u_{2}\right)}^{2}\cos^{2}\theta +{\left(r+u_{1}\right)}^{2}\sin^{2}\theta }}-r\\ 0\leq \theta \leq \frac{\pi }{2} & u_{1}\\ \frac{\pi }{2}\leq \theta \leq \pi & \frac{\left(r+u_{1})\cdot (r+u_{2}\right)}{\sqrt{{\left(r+u_{1}\right)}^{2}\cos^{2}\theta +{\left(r+u_{2}\right)}^{2}\sin^{2}\theta }}-r\\ \pi \leq \theta \leq \frac{3\pi }{2} & u_{2} \end{array}\right.$$

### 2.3. Radial Displacement Field and Corrosion Loss Model

The concrete cracking process comprises three stages [29]: the rust-free expansion stage, the concrete cover tensile stress stage, and the concrete cover cracking stage. The total quantity of steel corrosion products should be the sum of the corrosion products for each of the three stages. The capillary pores at the steel–concrete interface are of a uniform size around the reinforcement.

The thickness of the pore transition zone between the steel and concrete is *δ*_0_, the volume of the pore transition zone in unit length is 2π*rδ*_0_, and the corrosion volume of the reinforcement material in unit length is *V_s_*_1_. Thus, the volume *V*_1_ of the corrosion products is the sum of the pore transition zone volume and the reinforcement corrosion volume, as given by Equation (3).

(3) $$V_{1}=2\pi r\delta_{0}+V_{s1}$$

The volume of the corrosion product at the concrete cover tensile stress stage and the concrete cover cracking stage is *V*_2_. When the pore transition zone is filled with reinforcement corrosion products, the concrete around the reinforcement bar is displaced by the reinforcement rust expansion force. Thus, the expanded volume of the concrete around the reinforcement in unit length (*V_c_*) is calculated using the radial displacement around the reinforcement, as indicated by Equation (4). In both stages, the reinforcement corrosion volume in unit length is *V_s_*_2_. Thus, the volume *V*_2_ of the corrosion product is the sum of the expanded volume of the concrete and the corrosion volume of the reinforcement, as indicated by Equation (5).

(4) $$\mathrm{Non}-\mathrm{angular}\;\mathrm{area}\;\mathrm{reinforcement}:\;V_{c}=\frac{1}{2}\pi r(u_{1}+3u_{2})\;\mathrm{Angular}\;\mathrm{area}\;\mathrm{reinforcement}:\;V_{c}=\pi r(u_{1}+u_{2})$$

(5) $$V_{2}=V_{c}+V_{s2}$$

The total quantity *V_r_* of corrosion products is the sum of *V*_1_ and *V*_2_, as indicated by Equations (6) and (7). The expansion rate of the reinforcement corrosion products is *ρ*, and the relationship between *V_r_* and *V_s_* is given in Equation (8). The total corrosion volume of the reinforcement in unit length (*V_s_*) is given by Equation (9). The rate of reinforcement corrosion *ŋ* is given by Equation (10). The relationship between the reinforcement corrosion rate and the radial displacement is given by Equation (11).

*V*_*s*1_+ *V*_*s*2_= *V*_*s*_ (6)

(7) $$V_{r}=2\pi r\delta_{0}+V_{c}+V_{s}$$

(8) $$V_{r}=\rho V_{s}$$

(9) $$V_{s}=\frac{2\pi \delta_{0}+V_{c}}{\rho -1}$$

(10) $$\eta =\frac{V_{s}}{\pi r^{2}}=\frac{2\pi r_{0}+V_{c}}{(\rho -1)\pi r}$$

(11) $$\mathrm{Non}-\mathrm{angular}\;\mathrm{area}\;\mathrm{reinforcement}:\;\eta =\frac{4\delta_{0}+u_{1}+3u_{2}}{2(\rho -1)r}\;\mathrm{Angular}\;\mathrm{area}\;\mathrm{reinforcement}:\;\eta =\frac{2\delta_{0}+u_{1}+u_{2}}{(\rho -1)r}$$

Before corrosion expansion forms cracks in the concrete, the reinforcement corrosion is distributed in a semicircle near one side of the concrete protective layer, while the rust layer on the other side of the semicircle remains relatively small. Therefore, according to the literature [30], the relationship between the maximum radial displacement *u*_1_ and the minimum radial displacement *u*_2_ is given by Equation (12). The radial displacement equation is obtained by including the corrosion rate of the reinforcement (Equations (13) and (14)).

(12) $$u_{2}=u_{1}/30$$

Non-angular area reinforcement:

(13) $$u(\theta )=\left\{\begin{array}{cc} 0\leq \theta \leq \pi & \frac{r^{2}[1+2(\rho -1)\eta ]-4\delta_{0}r}{\sqrt{{\left[r+2r(\rho -1)\eta -4\delta_{0}\right]}^{2}\cos^{2}\theta +r^{2}\sin^{2}\theta }}-r\\ \pi \leq \theta \leq 2\pi & \left[2(\rho -1)r\eta -4\delta_{0}\right]/30 \end{array}\right.$$

Angular area reinforcement:

(14) $$u(\theta )=\left\{\begin{array}{cc} -\frac{\pi }{2}\leq \theta \leq 0 & \frac{r^{2}[1+2(\rho -1)\eta ]-4\delta_{0}r}{\sqrt{{\left[r+2r(\rho -1)\eta -4\delta_{0}\right]}^{2}\sin^{2}\theta +r^{2}\cos^{2}\theta }}-r\\ 0\;\leq \theta \leq \frac{\pi }{2} & 2(\rho -1)r\eta -4\delta_{0}\\ \frac{\pi }{2}\leq \theta \leq \pi & \frac{r^{2}[1+2(\rho -1)\eta ]-4\delta_{0}r}{\sqrt{{\left[r+2r(\rho -1)\eta -4\delta_{0}\right]}^{2}\cos^{2}\theta +r^{2}\sin^{2}\theta }}-r\;\\ \pi \leq \theta \leq \frac{3\pi }{2} & \left[2(\rho -1)r\eta -4\delta_{0}\right]/30 \end{array}\right.$$

Given the radial displacements *u*_1_ and *u*_2_, the corrosion rate and the quantity of steel reinforcement corrosion at the point when rust expansion causes the concrete to crack can be determined. According to the characteristics of the steel corrosion layer in the concrete, a mathematical model is obtained for the unequal radial displacement distribution field caused by the effect of steel corrosion on the concrete for both a non-angular and an angular area. The model elucidates the conditions underlying the additional stress field caused by steel corrosion expansion and allows finite element model analysis for predicting the concrete cracks. Moreover, the quantitative relationship between the section loss rate of the non-uniform corroded reinforcement and the radial displacement of the concrete around the steel bars is obtained by analyzing the entire corrosion process that results in concrete cracking.

## 3. Numerical Analysis Model

At present, there are two approaches for the analysis of the concrete protective layer cracking: the theoretical analytical approach and the experimental approach. The theoretical analysis mainly involves mechanics analysis and finite element analysis. The elastic mechanics analysis approach adopted by scholars involves the consideration of the elastic mechanics of a thick-walled cylinder model in which the expansion force of the corrosion product is assumed to be evenly distributed on the inner wall of the concrete cylinder. However, this assumption is unrealistic. An additional drawback of elastic mechanics analysis is the difficulty of accurate quantitative determination of the damage caused by concrete cracking. Although finite element analysis is a recommended and an accepted method for the analysis of concrete crack damage caused by steel corrosion, an alternative approach—experimentation—must also be considered. The experimental techniques include the impressed-current accelerated test method and the simulation test method. The former is generally widely used by researchers, as it has the advantage of relatively short test time. However, the corrosion results of the impressed-current accelerated test method correlate poorly with natural corrosion conditions. Meanwhile, the simulation test method is poor for simulating the uneven nature of reinforcement corrosion. Compared with the experimental method, the theoretical analysis method’s advantages of a short analysis period and low cost ensure that it plays an important role in the field of concrete crack damage research, including the establishment of a reinforcement corrosion model both before and at the time of concrete cracking [31,32,33] and the consideration of the quantitative relationship between the crack width and the corrosion rate. In the present study, the finite element software was used to investigate the cracking process of the concrete protective layer, caused by non-uniform corrosion and expansion of the steel reinforcement, and to identify the influencing factors.

### 3.1. Constitutive Relation and Failure Criterion of Concrete

**Figure 3:**
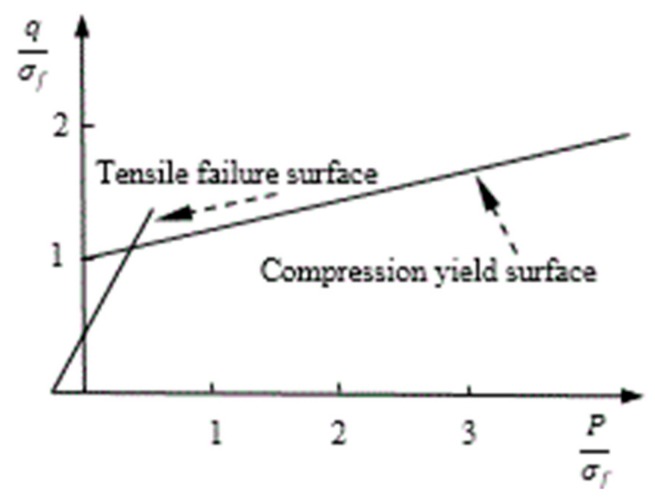
Concrete compression damage on the p–q plane.

**Figure 4:**
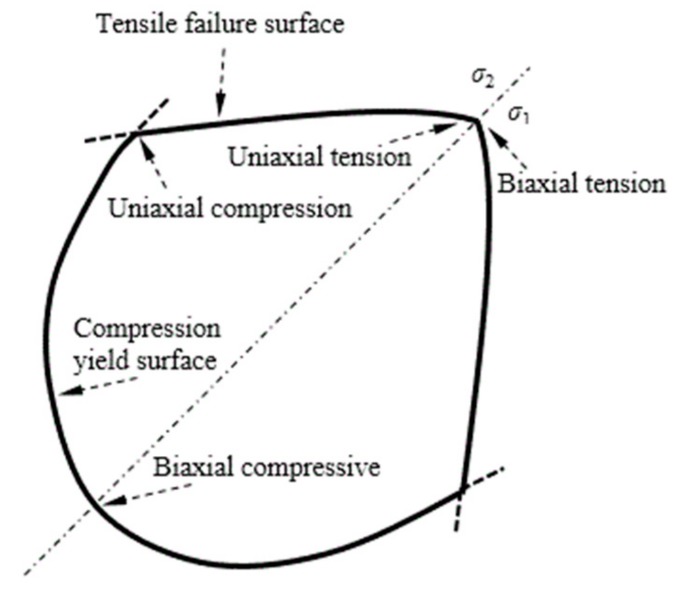
Yield and failure surfaces under biaxial stress.

The concrete constitutive relation refers mainly to the stress–strain relation of concrete subjected to uniaxial and multiaxial stresses. A concrete dispersion crack model is used to describe the nonlinear behavior of concrete caused by isotropic hardening under compression elastoplastic and elastic cracking tension. The compression yield surface and crack detection surface function on the p–q plane (compression damage) is shown in Figure 3, and the compressive yield surface and crack detection surface function under biaxial stress (tensile damage) is shown in Figure 4.

The nonlinear behavior of concrete under a load is expressed by the model partitions. The isotropic hardening elastoplastic model is used in the pressure–pressure model area and the elastic cracking model is used in other areas. The linear-elastic constitutive relation is adopted before concrete cracking, the crack detection surface (tensile damage) is determined by the stress state and fracture direction at the time of cracking, and the elastic constitutive relation is adopted after cracking. The equivalent hydrostatic pressure and equivalent deviatoric stress are used to express the compressive yield surface and crack detection (tensile damage), respectively. The “Failure Ratios” option defines the shape of the failure surfaces. Four values are necessary to express the model:

(1) The ratio of the biaxial ultimate compressive stress to the uniaxial ultimate compressive stress;
(2) The ratio of the uniaxial tensile ultimate failure stress to the uniaxial ultimate compressive stress;
(3) The ratio of the principal plastic strain component under the biaxial ultimate compressive stress to the plastic strain component corresponding to the uniaxial ultimate compressive stress; and
(4) The ratio of the principal tensile stress at cracking (the other two principal stresses combine to form the ultimate compressive stress) to the stress at the time of uniaxial tensile cracking.

The uniaxial compressive stress–strain relationship of the concrete was defined by the “concrete” option. The uniaxial compressive stress–strain relationship of GB50010-2010 [34] was adopted in this study. The relational model is given as follows:

(15) $$\sigma_{c}=f_{c}\cdot \left[\alpha_{a}\left(\frac{\varepsilon }{\varepsilon_{c}}\right)+\left(3-\alpha_{a}\right){\left(\frac{\varepsilon }{\varepsilon_{c}}\right)}^{2}+\left(\alpha_{a}-2\right){\left(\frac{\varepsilon }{\varepsilon_{c}}\right)}^{3}\right],$$

where *f_c_* represents the maximum uniaxial compressive stress, *ε_c_* represents the maximum uniaxial compression strain, and *α_a_* is the relevant parameter of the uniaxial compression stress–strain curve.

Cracking is one of the most important mechanical behaviors of concrete materials. The crack expression and the shape simulation after cracking are key parts of the model. In the numerical analysis of concrete, considering nonlinear behavior, the key problem is how to simulate concrete cracking. In this study, a dispersion cracking model with independent crack detection surfaces was adopted. Concrete cracks develop when the stress reaches the crack detection surface. The fracture directions are then stored and used for subsequent analysis and calculations. Because the damage theory is used, the fracture direction can affect the subsequent calculation once the crack is generated (crack may be open or closed). The subsequent failure behavior of the crack zone was simulated via “tensile hardening,” which must be defined in the concrete cracking model. There are two tension stiffening modes: the post-failure stress–strain relation and the fracture energy crack criterion. The post-failure stress–strain relation mode was used for plain concrete, but its calculation results exhibited mesh sensitivity. To solve this problem, the fracture energy crack criterion mode was adopted. The displacement of the crack surface was 0.05 mm when the stress was 0 in the model. The setting “TYPE=DISPLACEMENT” under the “TENSION STIFFENING” option in the model parameters was used to apply the initial displacement.

### 3.2. Finite Element Model 

An RC specimen with a section size of 200 mm × 200 mm was simulated using the finite element analysis model, in which the reinforcement was replaced with an equal diameter hole. C3D8R elements were used to simulate the concrete. C3D8R is a 3D eight-node linear hexahedral solid heat conduction unit that adopts the linear reduction integral of fine mesh division. Structural grid generation technology was adopted, and the grid size was 0.005 m. The meshes around the holes are more refined, in which the grid size was 2 mm. The density and shape of the mesh division must be specified for finite element calculations. A relatively convergent solution can be obtained via trial-and-error calculations with different meshing densities. The steel and concrete were assumed to be perfectly bonded. An “embedded” command was utilized to simulate the interaction between the concrete and steel. Moreover, a fixed connection at the end of the concrete cover was used as a boundary condition. In the cracking analysis, the main tasks included adding the mechanical property parameters of the materials, modifying the analysis types, adding displacement boundary conditions, applying loads, and outputting calculation results.

**Figure 5:**
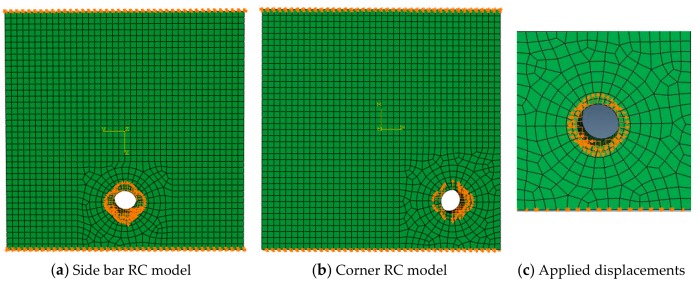
Reinforced concrete (RC) models.

The reinforcement corrosion was assumed to be uniform along the axial direction, such that the reinforcement corrosion expansion could be treated as a plane strain problem. The concrete angle bar model and concrete side bar model were simulated (Figure 5). The surrounding grid division was encrypted because of the stress concentration around the hole.

### 3.3. Model Parameters

The main factors influencing concrete cracking include the concrete strength, concrete cover thickness, rebar diameter, and steel position. The influencing factors considered in this study are presented in Table 1. These factors were combined in different ways to establish 54 models for the concrete cracking process.

### 3.4. Loading Method

The elliptical model described above was adopted to analyze the radial displacement field, and radial displacement was applied to simulate the uneven corrosion expansion of the reinforcement. Hinged bearings were set at the model boundary, such that the models were under tension in both the radial and axial directions. To facilitate analysis, initial defects and micro-cracks were ignored in the study. For the reinforcements in the angular and non-angular areas, the radial displacement of the concrete was applied according to Equations (13) and (14).

## 4. Analysis Results

### 4.1. Concrete Stress Analysis

A concrete model with C30 concrete strength, a cover thickness of 30 mm, and a steel diameter of 20 mm was analyzed.

#### 4.1.1. Single Steel Bar

**Figure 6:**
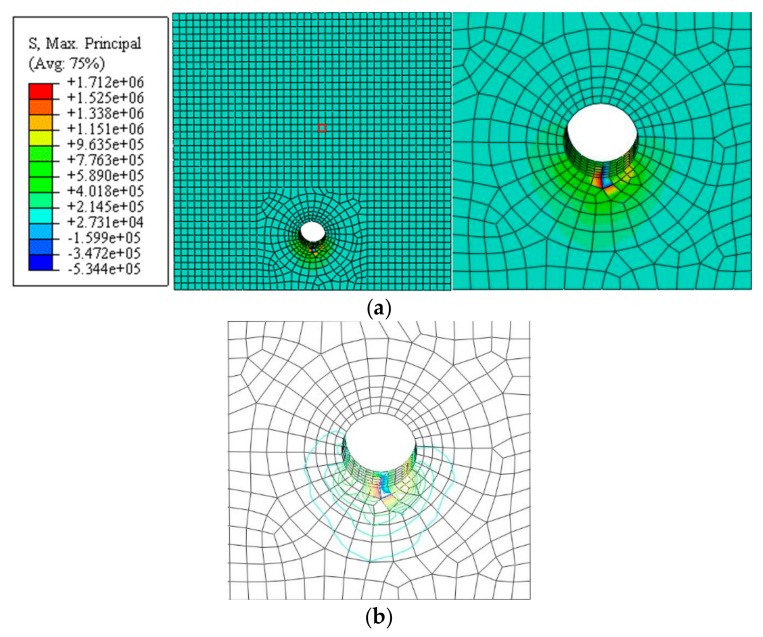
Non-angular area reinforcement (unit: Pa). (**a**) Nephogram of principal tensile stress. (**b**) Isoline map of principal tensile stress model.

**Figure 7:**
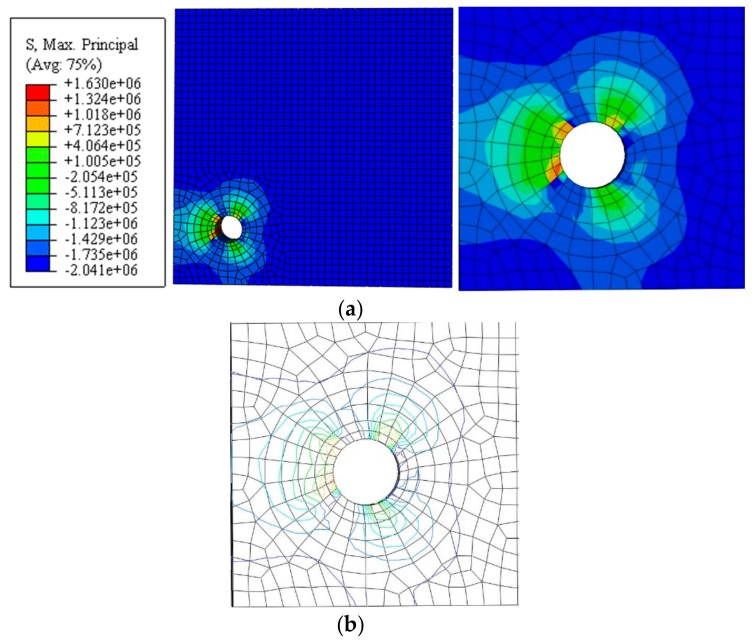
Angular area reinforcement (unit: Pa). (**a**) Nephogram of principal tensile stress. (**b**) Isoline map of principal tensile stress model.

The nephogram and isoline map of the principal tensile stress was obtained by applying a displacement value of *u*_1_ = 1 µm for the simulated stress distribution of uncracked concrete, as shown in Figure 6 and Figure 7 (unit: Pa). Here, the main tensile stress of the concrete at the edge of the hole was not evenly distributed. It decreased gradually from the edge to the outer region, whereas the main tensile stress at the edge of the outer surface increased.

#### 4.1.2. Multi-Reinforcement

**Figure 8:**
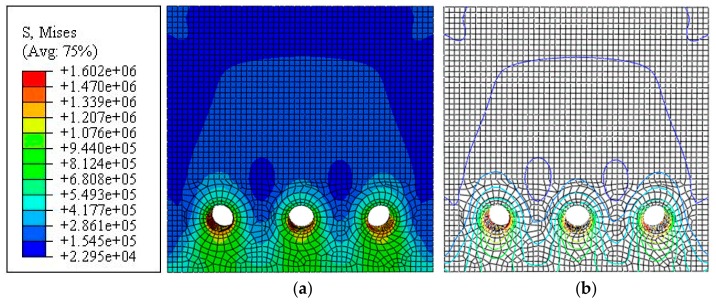
Multi-reinforcement: (**a**) Nephogram of principal tensile stress; (**b**) Isoline map of principal tensile stress model.

Here, three holes (one central hole and two angular holes, each with a diameter of 20 mm) were arranged evenly. Equal radial displacement was applied to obtain a nephogram and isoline map of the principal tensile stress, as shown in Figure 8. The figures indicate that the concrete stress was superimposed between adjacent steel bars, and that this stress was larger than that of the concrete protective layer. It can be inferred that when the steel bars rust simultaneously, the concrete cracks horizontally along the steel bars because of the stress superposition and even begins to peel off.

### 4.2. Radial Displacement of Reinforcement in Angular Area

#### 4.2.1. Numerical Analysis Results

**Figure 9:**
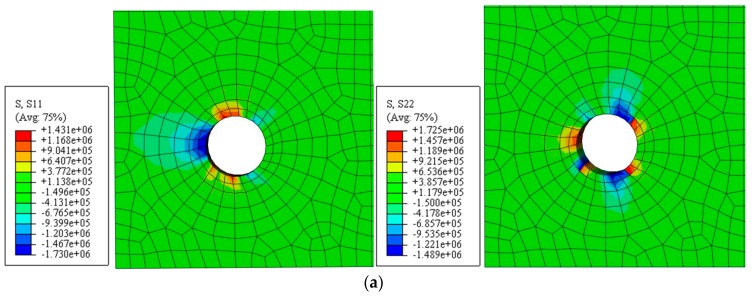

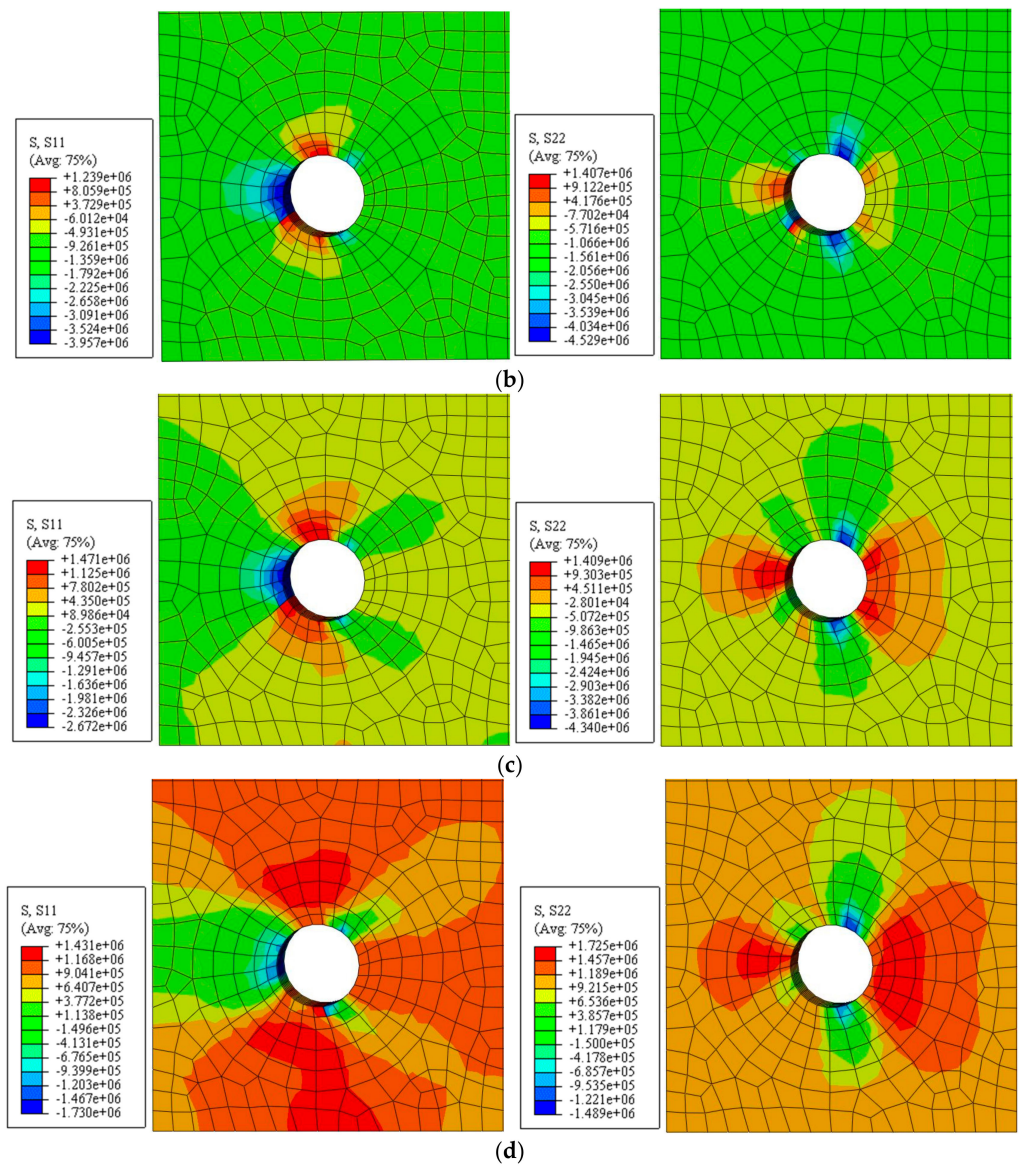
Horizontal and vertical components of the concrete stress (unit: Pa). (**a**) *u*_1_ = 2.5 µm; (**b**) *u*_1_ = 5 µm; (**c**) *u*_1_ = 6 µm; (**d**) *u*_1_ = 7.2 µm.

A concrete model with C30 concrete strength, a cover thickness of 30 mm, and a steel diameter of 20 mm was analyzed to obtain the critical radial displacement when concrete cracks. When a radial displacement of *u*_1_ = 2.5 µm was applied, cracks occurred in the concrete around the steel bar. When a radial displacement of *u*_1_ = 5 µm was applied, the cracks spread in all directions. When a radial displacement of *u*_1_ = 6 µm was applied, cracks appeared in the concrete protective layer. When a radial displacement of *u*_1_ = 7.2 µm was applied, cracks penetrated the concrete protective layer. The horizontal and vertical components of the concrete stress values for the cracked surface are shown in Figure 9, and the results of the numerical analysis of the concrete cracking caused by the non-uniform corrosion of the reinforcing steel in the angular region are presented in Table 2.

#### 4.2.2. Analysis of Influencing Factors

(1) Concrete Strength Grade

**Figure 10:**
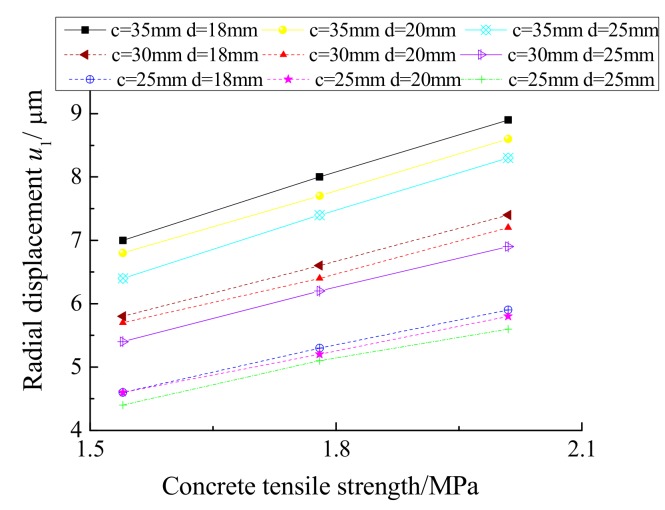
Radial displacement *u*_1_ and the tensile strength of the concrete.

According to Table 2, the relationship between the radial displacement *u*_1_ and the tensile strength of the concrete when the concrete protective layer cracks was established, as shown in Figure 10. The radial displacement *u*_1_ increases gradually as the tensile strength increases when the concrete cover thickness and rebar diameter remain constant.

(2) Concrete Cover Thickness and Rebar Diameter

**Figure 11:**
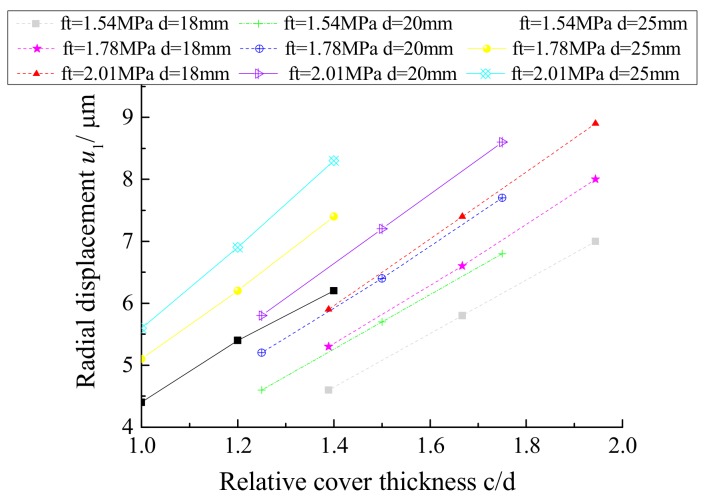
Radial displacement *u*_1_ and relative cover thickness *c*/*d*.

Table 2 presents the relationship between the radial displacement *u*_1_ and the ratio of the concrete cover thickness to the rebar diameter when the concrete protective layer cracks, as shown in Figure 11. It can be concluded that the radial displacement *u*_1_ increases gradually as the relative cover thickness increases when the tensile strength of the concrete remains constant.

(3) Regression Analysis Data

Factor analysis results indicated that the regression formula considering the critical radial displacement, concrete strength, cover thickness, and rebar diameter could be obtained using multiple logistic regression analyses. The regression model was established using the fitting function of Equation (16), as given in Equation (17).

(16) $$u_{1}=k\cdot f_{t}{}^{A}\cdot {\left(1+c/d\right)}^{B}\cdot d^{C}$$

(17) $$u_{1}=0.0338\cdot f_{t}{}^{0.92}\cdot {\left(1+c/d\right)}^{2}\cdot d^{}$$

where, *u*_1_ represents the critical radial displacement, *f_t_* represents the tensile strength of the concrete, *c* represents the cover thickness, and *d* represents the rebar diameter.

### 4.3. Radial Displacement of Reinforcement in Non-Angular Area

#### 4.3.1. Numerical Analysis Results

**Figure 12:**
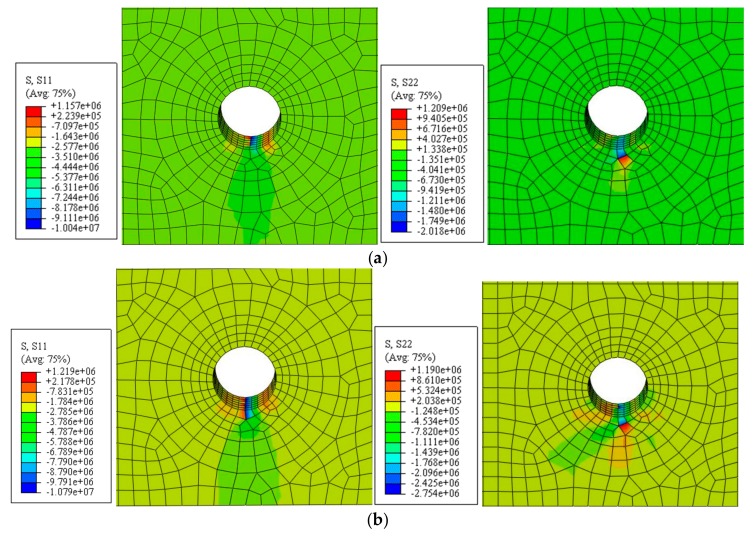

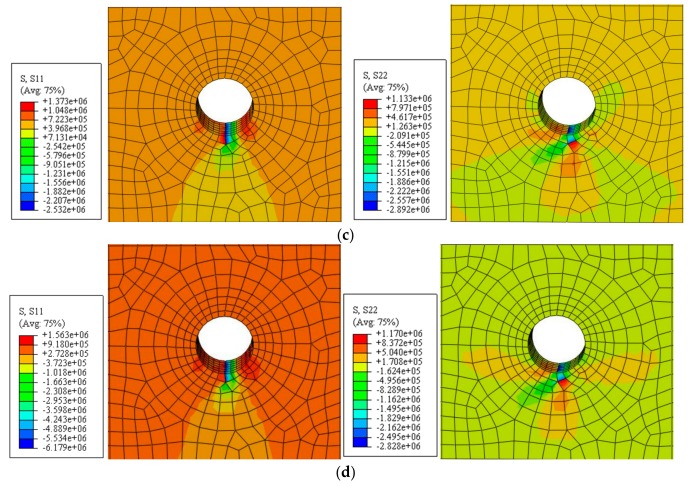
Horizontal and vertical components of the concrete stress (unit: Pa): (**a**) *u*_1_ = 4.5 µm; (**b**) *u*_1_ = 7.4 µm; (**c**) *u*_1_ = 11.3 µm; (**d**) *u*_1_ = 11.9 µm.

A concrete model with C30 concrete strength, a cover thickness of 30 mm, and a steel diameter of 20 mm were analyzed to determine the critical radial displacement during concrete cracking. When a radial displacement of *u*_1_ = 4.5 µm was applied, cracks occurred in the concrete around the steel bar. When a radial displacement of *u*_1_ = 7.4 µm was applied, the cracks spread in all directions. When a radial displacement of *u*_1_ = 11.3 µm was applied, cracks appeared in the concrete protective layer. When a radial displacement of *u*_1_ = 11.9 µm was applied, the cracks penetrated the concrete protective layer. The horizontal and vertical components of the concrete stress on the cracked surface are shown in Figure 12. The numerical analysis results for the concrete cracking caused by the non-uniform corrosion of the reinforcing steel in the non-angular area are presented in Table 3.

#### 4.3.2. Analysis of Influencing Factors

(1) Concrete Strength Grade

**Figure 13:**
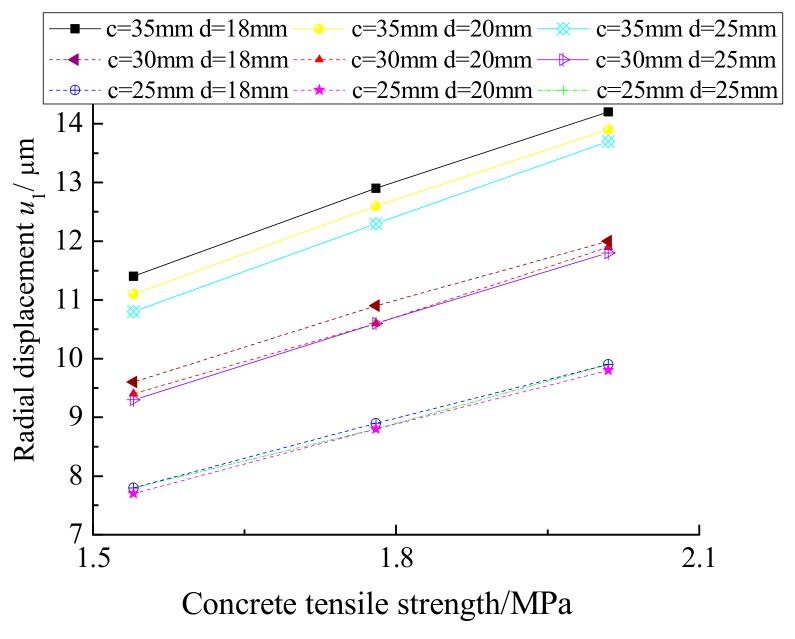
Radial displacement *u*_1_ and the tensile strength of the concrete.

The relationship between the radial displacement *u*_1_ and the tensile strength of the concrete when the concrete protective layer cracks can be determined from Table 3 and is shown in Figure 13. The radial displacement *u*_1_ increases gradually as the tensile strength increases when the concrete cover thickness and rebar diameter remain constant.

(2) Relative Concrete Cover Thickness

**Figure 14:**
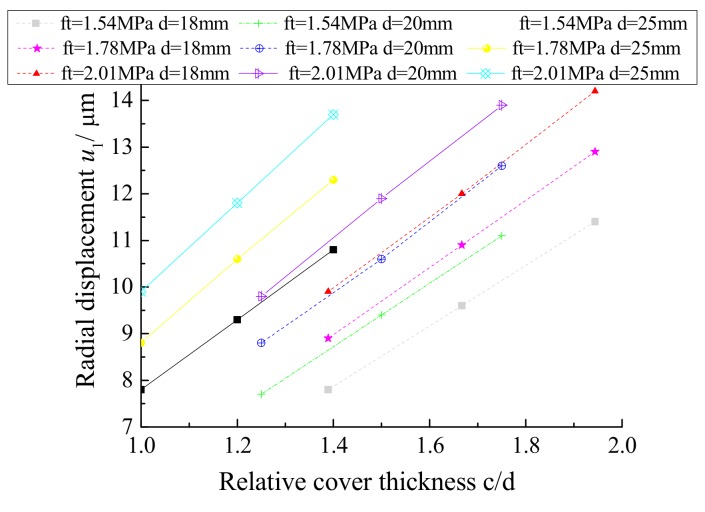
Radial displacement *u*_1_ and relative cover thickness *c*/*d*.

The relationship between the radial displacement *u*_1_ and the ratio of the concrete cover thickness to the rebar diameter when the concrete protective layer cracks can be determined from Table 3 and is shown in Figure 14. The radial displacement *u*_1_ increases gradually as the relative cover thickness increases when the tensile strength of the concrete remains constant.

(3) Regression Analysis Data

The critical radial displacement of a non-angular reinforcement area is larger than that of an angular area reinforcement area, as indicated by Table 4. The ratio of the former to the latter is approximately 1.68. Therefore, a reinforcement-position correction parameter was adopted for proper consideration of the effect of a non-angular location. The regression model for the critical radial displacement *u*_1_ of the protective layer caused by the non-uniform rust in the non-angular area was corrected in accordance with Equation (18).

(18) $$u_{1}=0.0568\cdot f_{t}{}^{0.92}\cdot {\left(1+c/d\right)}^{2}\cdot d^{}$$

Here, *u*_1_ represents the critical radial displacement, *f_t_* represents the tensile strength of the concrete, *c* represents the cover thickness, and *d* represents the rebar diameter.

### 4.4. Reinforcement Corrosion Rate Model

Equations (17) and (18), respectively, were substituted into Equation (11), and the steel corrosion rate models for the non-angular and angular areas are given in Equation (19). The regression mathematical model of the steel corrosion rate was obtained using the reinforcement-position correction parameters, as indicated by Equation (20). The radial displacement equation was obtained by including the reinforcement corrosion rate, as indicated by Equations (21) and (22).

(19) $$\mathrm{Non}-\mathrm{angular}\;\mathrm{area}\;\mathrm{reinforcement}\;\eta =\frac{4\delta_{0}+0.063\cdot f_{t}{}^{0.92}\cdot {\left(1+c/d\right)}^{2}\cdot d^{}}{(\rho -1)d}\;\mathrm{Angular}\;\mathrm{area}\;\mathrm{reinforcement}\;\eta =\frac{4\delta_{0}+0.07\cdot f_{t}{}^{0.92}\cdot {\left(1+c/d\right)}^{2}\cdot d^{}}{(\rho -1)d}$$

(20) $$\eta =\frac{4\delta_{0}+k\cdot 0.07\cdot f_{t}{}^{0.92}\cdot {\left(1+c/d\right)}^{2}\cdot d^{}}{(\rho -1)d}$$

Here, *δ*_0_ represents the thickness of the pore transition zone between the steel and concrete, *ρ* represents the expansion rate of the reinforcement corrosion products, and *k* represents the reinforcement-position correction parameter, which is 1 in the angular area and 0.9 for the reinforcement in the non-angular region.

Non-angular area reinforcement:

(21) $$u(\theta )=\left\{\begin{array}{cc} 0\leq \theta \leq \pi & \frac{r^{2}+8\delta_{0}r^{2}/d+0.126r^{2}f_{t}^{0.92}{\left(1+c/d\right)}^{2}-4\delta_{0}r}{\sqrt{{\left[r+8\delta_{0}r/d+0.126rf_{t}^{0.92}{\left(1+c/d\right)}^{2}-4\delta_{0}\right]}^{2}\cos^{2}\theta +r^{2}\sin^{2}\theta }}-r\\ \pi \leq \theta \leq 2\pi & 0.267\delta_{0}r/d+0.004rf_{t}^{0.92}{\left(1+c/d\right)}^{2}-0.133\delta_{0} \end{array}\right.$$

Angular area reinforcement:

(22) $$u(\theta )=\left\{\begin{array}{cc} -\frac{\pi }{2}\leq \theta \leq 0 & \frac{r^{2}+8\delta_{0}r^{2}/d+0.126r^{2}f_{t}^{0.92}{\left(1+c/d\right)}^{2}-4\delta_{0}r}{\sqrt{{\left[r+8\delta_{0}r/d+0.126rf_{t}^{0.92}{\left(1+c/d\right)}^{2}-4\delta_{0}\right]}^{2}\cos^{2}\theta +r^{2}\sin^{2}\theta }}-r\\ 0\leq \theta \leq \frac{\pi }{2} & 8\delta_{0}r/d+0.126rf_{t}^{0.92}{\left(1+c/d\right)}^{2}-4\delta_{0}\\ \frac{\pi }{2}\leq \theta \leq \pi & \frac{r^{2}+8\delta_{0}r^{2}/d+0.126r^{2}f_{t}^{0.92}{\left(1+c/d\right)}^{2}-4\delta_{0}r}{\sqrt{{\left[r+8\delta_{0}r/d+0.126rf_{t}^{0.92}{\left(1+c/d\right)}^{2}-4\delta_{0}\right]}^{2}\sin^{2}\theta +r^{2}\cos^{2}\theta }}-r\\ \pi \leq \theta \leq \frac{3\pi }{2} & 0.267\delta_{0}r/d+0.004rf_{t}^{0.92}{\left(1+c/d\right)}^{2}-0.133\delta_{0} \end{array}\right.$$

### 4.5. Test Verification

**Figure 15:**
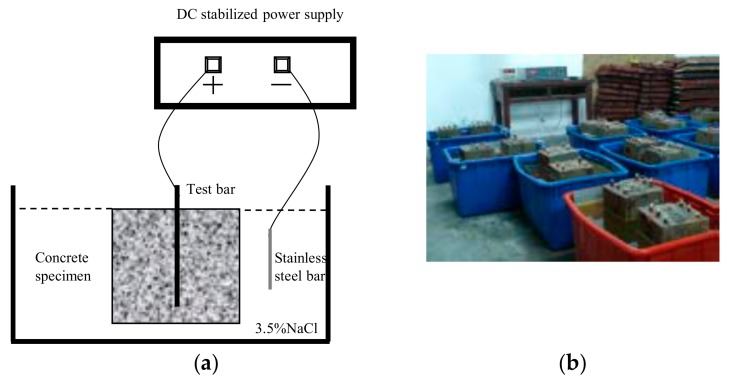
Field acceleration test. (**a**) Testing device. (**b**) Concrete sample.

To validate the formula, a 300 mm × 300 mm × 500 mm RC specimen was tested to determine the rate of steel corrosion. The cementing material used in the experiment was P.O42.5 ordinary Portland cement. Ordinary concrete fine aggregates were made of river sand with a fineness modulus of 2.4. Coarse aggregates were made of basalt gravel with a particle size of 5–20 mm. Both the fine and coarse aggregates satisfied the requirements of the aggregate grading curve. The water-reducing admixture was a polycarboxylic acid superplasticizer with a water reduction rate of 25%–30% (mass fraction), and the mixing water was tap water. The tensile strength of concrete was obtained via a splitting test involving cubic specimens. The splitting tensile strength of the concrete was evaluated for three cubic test blocks with dimensions of 150 mm × 150 mm × 150 mm. To reduce the influence of pouring factors, the same batch of sample curing was adopted. The mixture ratios of the C30 concrete are presented in Table 5. The properties of the concrete test blocks are presented in Table 6. The composition and mechanical properties of the reinforcement are presented in Table 7. The corrosion rate of the steel reinforcement was evaluated via an electric field-accelerated corrosion method. RC specimens were placed in a 3.5% NaCl solution. Stainless steel in the solution as an auxiliary electrode (cathode) was connected to the negative electrode of a stabilized current meter. The rust reinforcement in the concrete test block served as the anode and was connected to the positive electrode of the stabilized current meter, as shown in Figure 15. The corrosion current density was fixed at 2 A/mm^2^. The crack width was monitored using a crack width meter. When it was found that the crack width of the specimen surface along the longitudinal reinforcement was 0.10 mm, the broken specimen was removed for rust removal and weighing. The weight loss rate of the reinforcement was the corrosion rate when the protective layer cracked.

After the accelerated corrosion tests, the specimens were cut into slices to analyze the corrosion pattern by microscopy in the inner reinforced mortar. A half sample was used to analyze the corrosion process by using inter-correlation image technique at the upper surface. The rust layer on the mortar was analyzed by using a CCD camera. According to a microscopic test, *δ*_0_ was 12.5 µm, and *ρ* was 2. The mathematical regression model for the steel corrosion rate was simplified to Equation (23). The corrosion rate of the steel reinforcement was evaluated using the weightlessness method. The test and numerical simulation results are presented in Table 8.

(23) $$\eta =0.05d^{-1}+k\cdot 0.07\cdot f_{t}{}^{0.92}\cdot {\left(1+c/d\right)}^{2}$$

From this table, the calculated results were close to the experimental results, the error between them was <5%. The error occurred because the phenomenon of the rust products filling the crack gap and the creep effect of the concrete were not considered in the numerical simulation.

## 5. Conclusions

As the corrosion of steel bars seriously affects the durability of concrete structures, the analysis of the concrete cracking process caused by reinforcement corrosion has important research significance. For the non-uniform corrosion of steel bars, uneven rust expansion displacement was used to simulate the cracking process of the concrete protective layer using numerical simulation.

(1) According to the occurrence of non-uniform corrosion and the characteristics of the rust layer, the unequal radial displacement distribution function of concrete around both an angular and a non-angular bar was deduced.
(2) The stress field distribution of the concrete protective layer caused by steel corrosion was summarized, and the presence of adjacent steel bars caused a stress superposition of the concrete between the steel bars.
(3) The formula for calculating the critical radial displacement was obtained at the point when cracking begun and used to predict the corrosion ratio of the covering concrete.
(4) The mathematical model of the radial displacement at the time cracking occurred was obtained, which is related to the tensile strength of the concrete, the relative protective layer and diameter of rebar.
(5) The correctness of the non-uniform rust calculation model was verified by comparing the results of the numerical analysis with the experimental results.

## Figures and Tables

**Table 1:** Material parameter.

Number	Rebar Diameter/mm	Cover Thickness/mm	Tensile Strength/MPa	Reinforced Location
1	18	25	1.54	Side reinforcement
2	20	30	1.78	Corner reinforcement
3	25	35	2.01	-

**Table 2:** Critical radial displacement of the corner reinforcement.

Num.	Tensile Strength/MPa	Rebar Diameter/mm	Cover Thickness/mm	Radial Displacement *u*_1_/µm	Num.	Tensile Strength/MPa	Rebar Diameter/mm	Cover Thickness/mm	Radial Displacement *u*_1_/µm
1	1.54	18	25	4.6	15	1.78	20	35	7.7
2	1.54	18	30	5.8	16	1.78	25	25	5.1
3	1.54	18	35	7.0	17	1.78	25	30	6.2
4	1.54	20	25	4.6	18	1.78	25	35	7.4
5	1.54	20	30	5.7	19	2.01	18	25	5.9
6	1.54	20	35	6.8	20	2.01	18	30	7.4
7	1.54	25	25	4.4	21	2.01	18	35	8.9
8	1.54	25	30	5.4	22	2.01	20	25	5.8
9	1.54	25	35	5.9	23	2.01	20	30	7.2
10	1.78	18	25	5.3	24	2.01	20	35	8.6
11	1.78	18	30	6.6	25	2.01	25	25	5.6
12	1.78	18	35	8.0	26	2.01	25	30	6.9
13	1.78	20	25	5.2	27	2.01	25	35	8.3
14	1.78	20	30	6.4					

**Table 3:** Critical radial displacement of the non-angle reinforcement.

Num.	Tensile Strength/MPa	Rebar Diameter/mm	Cover Thickness/mm	Radial Displacement *u*_1_/µm	Num.	Tensile Strength/MPa	Rebar Diameter/mm	Cover Thickness/mm	Radial Displacement *u*_1_/µm
1	1.54	18	25	7.8	15	1.78	20	35	12.6
2	1.54	18	30	9.6	16	1.78	25	25	8.8
3	1.54	18	35	11.4	17	1.78	25	30	10.6
4	1.54	20	25	7.7	18	1.78	25	35	12.3
5	1.54	20	30	9.4	19	2.01	18	25	9.9
6	1.54	20	35	11.1	20	2.01	18	30	12.0
7	1.54	25	25	7.8	21	2.01	18	35	14.2
8	1.54	25	30	9.3	22	2.01	20	25	9.8
9	1.54	25	35	10.8	23	2.01	20	30	11.9
10	1.78	18	25	8.9	24	2.01	20	35	13.9
11	1.78	18	30	10.9	25	2.01	25	25	9.9
12	1.78	18	35	12.9	26	2.01	25	30	11.8
13	1.78	20	25	8.8	27	2.01	25	35	13.7
14	1.78	20	30	10.6					

**Table 4:** Ratio analysis of critical radial displacement.

Num.	Non-Angular Radial Displacement/µm	Angular Radial Displacement/µm	Ratio	Num.	Non-Angular Radial Displacement/µm	Angular Radial Displacement/µm	Ratio
1	7.8	4.6	1.70	15	12.6	7.7	1.64
2	9.6	5.8	1.66	16	8.8	5.1	1.73
3	11.4	7.0	1.63	17	10.6	6.2	1.71
4	7.7	4.6	1.67	18	12.3	7.4	1.66
5	9.4	5.7	1.65	19	9.9	5.9	1.68
6	11.1	6.8	1.63	20	12.0	7.4	1.62
7	7.8	4.4	1.77	21	14.2	8.9	1.60
8	9.3	5.4	1.72	22	9.8	5.8	1.69
9	10.8	5.9	1.83	23	11.9	7.2	1.65
10	8.9	5.3	1.68	24	13.9	8.6	1.62
11	10.9	6.6	1.65	25	9.9	5.6	1.77
12	12.9	8.0	1.61	26	11.8	6.9	1.71
13	8.8	5.2	1.69	27	13.7	8.3	1.65
14	10.6	6.4	1.66				

**Table 5:** Contents of the concrete mixture (kg/m^3^).

Number	Cement	Water	Water Reducer	Water–Binder Ratio	Fine Aggregate	Coarse Aggregate
C30	381	171	1.48	0.45	718	1068

**Table 6:** Concrete properties.

Number	Rebar Diameter/mm	Cover Thickness/mm	Tensile Strength/MPa
1	18	25	2.01
2	18	30	2.01
3	18	35	2.01
4	20	30	2.01
5	25	30	2.01

**Table 7:** Composition and mechanical properties of the reinforcement.

Steel Type	Composition/%						Mechanical Properties			
	C	Si	Mn	P	S	Fe	Yield Strength/MPa	Extension Strength/MPa	Elongation	Yield Strength Ratio
HRB400	0.25	0.31	1.08	0.015	0.025	other	480	630	1.31	1.20

**Table 8:** Results of the test and numerical simulation.

Number	Steel Corrosion Rate/%		
	Numerical Simulation Method	Test	Error
1	0.686	0.715	4.27
2	0.854	0.890	4.18
3	1.041	1.089	4.62
4	0.751	0.780	3.80
5	0.582	0.605	3.97
